# Genetic basis of maturity time is independent from that of flowering time and contributes to ecotype differentiation in common buckwheat (*Fagopyrum esculentum* Moench)

**DOI:** 10.1186/s12870-022-03722-6

**Published:** 2022-07-21

**Authors:** Ryoma Takeshima, Shiori Yabe, Katsuhiro Matsui

**Affiliations:** 1 Institute of Crop Science, National Agriculture and Food Research Organization (NARO), Kannondai 2-1-2, Tsukuba, Ibaraki, 305-8518 Japan; 2 Graduate School of Life and Environmental Science, University of Tsukuba, Tennodai 1-1-1, Tsukuba, Ibaraki, 305-8572 Japan

**Keywords:** Common buckwheat, Ecotype, GRAS-Di, Maturity time, Photosensitivity

## Abstract

**Background:**

Common buckwheat is considered a quantitative short-day plant and is classified into the autumn (highly photoperiod sensitive), summer (weakly photoperiod sensitive), and intermediate ecotype. Understanding ecotype differentiation is essential for adaptive expansion and maximizing yield. The genetic analysis for ecotype has focused on photoperiod-dependent flowering time, whereas post-flowering traits such as seed set and maturity time might also regulate ecotype differentiation.

**Results:**

A field experiment revealed that ecotype differentiation is mainly defined by the timing of seed set and maturation, whereas flowering time is less relevant. Thus, we focused on maturity time as a trait that defines the ecotype. To detect QTLs for maturity time, we developed two F_2_ populations derived from early × late-maturing accessions and intermediate × late-maturing accessions. Using genotyping by random amplicon sequencing–direct analysis, we generated a high-density linkage map. QTL analysis detected two major QTLs for maturity time, one in each F_2_ population. We also detected QTLs for flowering time at loci different from maturity time QTLs, which suggests that different genetic mechanisms regulate flowering and maturity. Association analysis showed that both QTLs for maturity time were significantly associated with variations in the trait across years.

**Conclusions:**

Maturity time appeared to be more suitable for explaining ecotype differentiation than flowering time, and different genetic mechanisms would regulate the timing of flowering and maturation. The QTLs and QTL-linked markers for maturity time detected here may be useful to extend the cultivation area and to fine-tune the growth period to maximize yield in buckwheat.

**Supplementary Information:**

The online version contains supplementary material available at 10.1186/s12870-022-03722-6.

## Background

Common buckwheat (*Fagopyrum esculentum* Moench; 2*n* = 2*x* = 16) is an outcrossing pseudo-cereal widely grown from Asia to Europe, North America, and South Africa [1]. Buckwheat has a short growing period (generally 70–90 days) and can grow in a wide range of environmental conditions such as cool climates, high elevations, well-drained sandy soils, marginal lands, and acidic soils (pH < 5) [2–5]. Its seeds contain high levels of starch (without gluten) and high-quality protein with a well-balanced amino acid profile, and health-promoting antioxidative, antihypertensive, and anti-obesity compounds [6–9]. Expanding the cultivation area and increasing the production of this valuable orphan crop may contribute to future food security. Although buckwheat is grown in a wide range of latitudes, the cultivation areas and seasons of each genotype are strictly limited. Determining the genetic mechanisms of adaptability of each genotype is the first step toward expanding cultivation areas and maximizing yield.

The adaptability of buckwheat is thought to result from the differentiation of each cultivar into ecotypes appropriate for their cultivation areas [10]. Buckwheat is considered a quantitative short-day (SD) plant and is classified into three ecotypes according to their cultivation areas and seasons: autumn ecotype (highly photoperiod sensitive), summer ecotype (weakly photoperiod sensitive), and intermediate ecotype [11]. Autumn-ecotype cultivars have late flowering, low seed-set ratio, continuous flowering, and vigorous vegetative growth, late maturation, and much lower yield under summer (long-day, LD) cultivation than under autumn cultivation. In contrast, summer-ecotype cultivars have early flowering, high seed-set ratio, and early maturation under summer cultivation, but their yield is somewhat less under autumn cultivation than summer cultivation [12–16]. Understanding ecotype differentiation is essential for adaptive expansion and yield maximization because the mismatch between ecotype and environment considerably decreases yield potential. However, genetic analysis of buckwheat ecotypes has been limited to that of flowering time in response to day length [17, 18]. The response of flowering time to photoperiod has often been studied to evaluate buckwheat ecotype, but day length appears to affect not only flowering time but also post-flowering reproductive development. For example, in autumn-ecotype cultivars and some intermediate-ecotype cultivars, summer cultivation increases the number of malformed flowers, inhibits pistil development, leads to abnormal embryo sacs after pollination, and decreases pollen fertility and the ability to set seed [14, 19–22]. Thus, to understand the genetic mechanism of ecotype differentiation, it is necessary to reveal the effects of day length not only on flowering time but also on seed set and maturation.

The genetic analysis of common buckwheat is still challenging because of its heteromorphic self-incompatibility (SI) with two types of floral architecture: thrum (short style) and pin (long style) [23]. To allow genetic analysis in F_2_ populations, we previously used the self-compatible common buckwheat line ‘Kyukei SC7’ (KSC7), developed by introducing the self-compatibility (SC) allele from a wild relative, *F. homotropicum*, and identified a genetic region related to preharvest sprouting tolerance [24]. We also developed a genome-wide marker set based on the Single Nucleotide Polymorphism (SNP) information of parental lines. However, the usability of this marker set depends on the genetic structure of parents, and a more global marker set is needed. The genotyping by random amplicon sequencing–direct (GRAS-Di) system, a derivative of amplicon sequencing technology and used random primers for PCR amplification, can identify many markers covering all chromosomes even in a genetic population with small genetic variation [25]. Because the GRAS-Di system mainly provides information for dominant genotyping, GRAS-Di combined with a mapping-based genotyping method applied to a high-quality reference genome is reportedly efficient for obtaining information for co-dominant genotyping, constructing a linkage map, and detecting quantitative trait loci (QTLs) [26, 27]. Although a draft genome sequence (N50 = 25 kb, 387,594 scaffolds) [28] and a recently published reference genome of the Russian cultivar ‘Dasha’ (N50 = 188 kb, 85,178 scaffolds) [29] are available for buckwheat, the large number of scaffolds hampers mapping-based analysis.

Here, we investigated the genetic loci associated with maturity time as a trait that defines the ecotype under natural LD conditions. In our cultivation condition, ecotype differentiation is defined mainly by the ability to set seed and by maturity time, whereas flowering time is less relevant. Using F_2_ segregating populations, we developed a high-density genetic linkage map by GRAS-Di analysis via re-estimation of co-dominant marker genotypes. Using this map, we detected major QTLs for maturity time. These QTLs were located at loci different from those of the flowering time QTLs, suggesting that the photoperiod responses of maturity time and flowering time would have different mechanisms.

## Results

### Photoperiod response of world buckwheat germplasms under natural long-day conditions

**Fig. 1:**
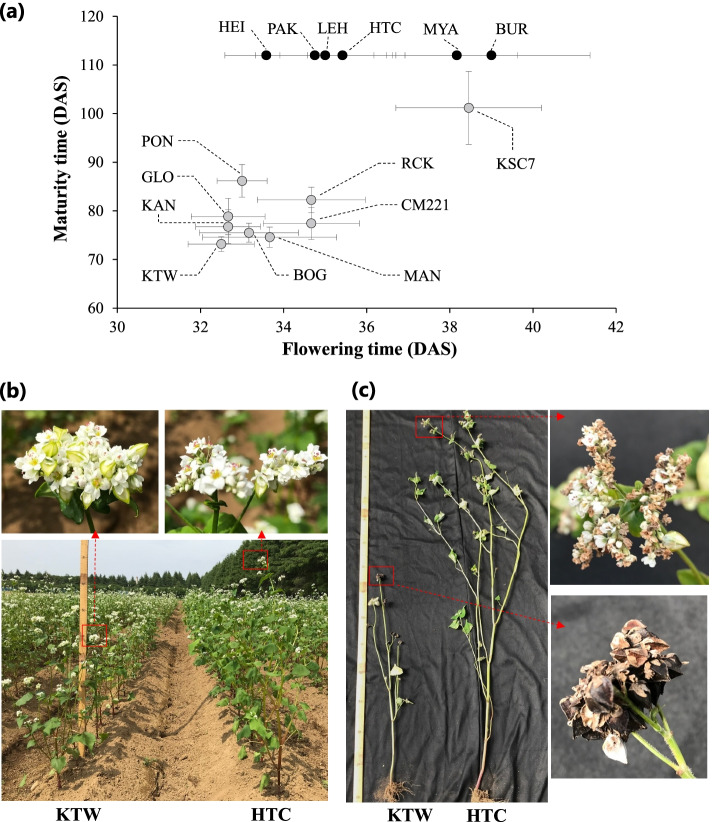
Variation of photoperiod response among world buckwheat germplasms. **a** First flowering time and maturity time of 15 germplasms under natural long-day conditions. Detailed information on each germplasm is provided in Table S7. Germplasms shown as black circles did not mature until the end of the experiment (112 days after sowing, DAS). **b** Growth patterns of ‘Kitawase-soba’ (KTW) and ‘Hitachi-akisoba’ (HTC) at 40 DAS. **c** Maturation patterns of KTW and HTC at 70 DAS

Maturity time varied more widely across germplasms than did flowering time (Fig. 1a): average flowering time ranged from 32.5 to 39.0 days after sowing (DAS), whereas average maturity time ranged from 73.2 to 101.2 DAS among nine germplasms, and six germplasms did not mature until the end of the experiment (112 DAS). These six germplasms continued vigorous vegetative growth and flowering, but seed set was rarely successful and flowers were aborted (Fig. 1b and c). Flowering time did not allow to predict maturity time; e.g., accession HEI flowered earlier than MAN, CM221, RCK, and KSC7, but did not mature until 112 DAS. This result suggested that flowering time is insufficient to determine buckwheat suitability for a particular growing environment, and the ability to set seed and maturity time might be more appropriate determinants of the ecotype. To determine the genetic mechanism for maturity time, we selected three parental lines: ‘Kitawase-soba’ (KTW; early-maturing), ‘Ruchi-king’ (RCK; intermediate-maturing), and SC line KSC7 (late-maturing).

### Distribution of maturity time and flowering time in F_2_ segregating populations

**Fig. 2:**
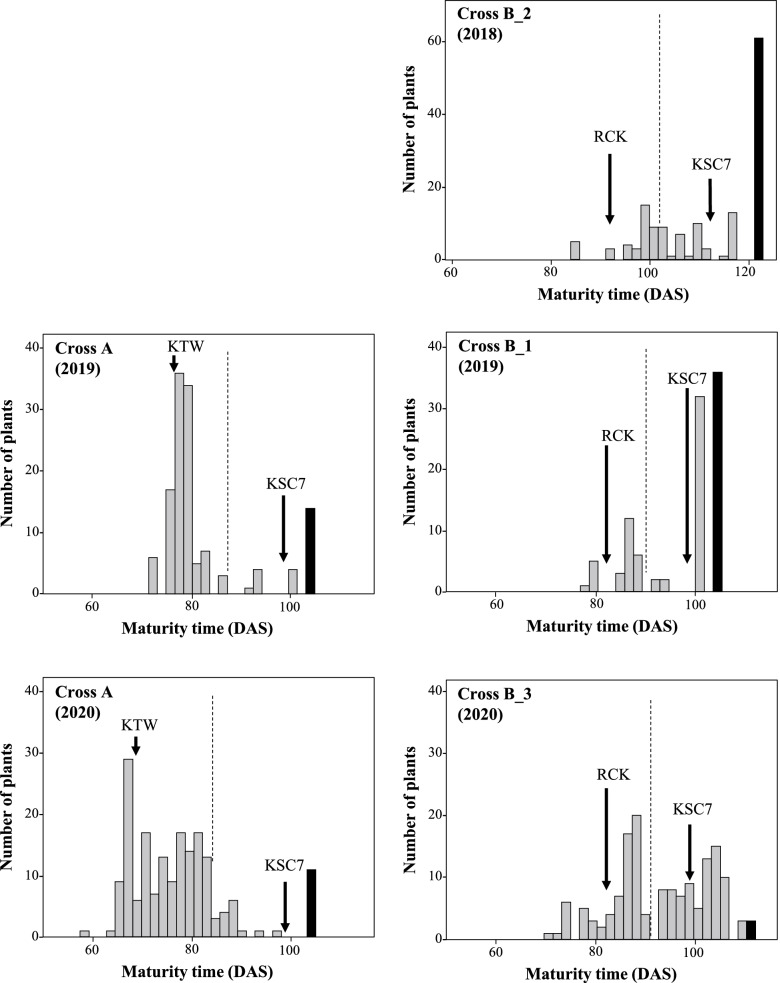
Segregation of maturity time in F_2_ progenies of crosses between ‘Kitawase-soba’ (KTW) and ‘Kyukei SC 7’ (KSC7) (Cross A), and between ‘Ruchi-king’ (RCK) and KSC7 (Cross B) under natural long-day conditions. Black bars, individuals that did not mature until the end of the experiment (122 DAS in Cross B_2, and 105 DAS in Crosses A, B_1, 112 DAS in Cross B_3). Arrows indicate mean values of maturity time in parents. Dotted vertical lines indicate the average of two parents. DAS, days after sowing

We developed four F_2_ populations derived from crosses between Cross A (KTW/KSC7), Cross B_1 (RCK_1/KSC7), Cross B_2 (RCK_2/KSC7), and Cross B_3 (RCK_3/KSC7). The segregation pattern of maturity time of Cross A tended to be bi-modal; most plants matured before the average maturity time of the parents (87.3 DAS in 2019 and 83.7 DAS in 2020) (Fig. 2). In Crosses B_1 and B_2, the segregation pattern also tended to be bi-modal but leaned toward late maturity. On the other hand, the segregation pattern in Cross B_3 showed a normal distribution. Because buckwheat is an outbreeding species, the genotypes differ even within the same cultivar. Flowering time showed normal distributions in all crosses (Fig. S1). The correlation coefficient between flowering time and maturity time was 0.392 in Cross A (2019), 0.506 in Cross A (2020), 0.165 in Cross B_1 (2019), − 0.080 in Cross B_2 (2018), and 0.365 in Cross B_3 (2020) (Fig. S2). The absence of a strong correlation between flowering time and maturity time suggested that different genetic loci regulate the timing of flowering and maturation.

### Development of a GRAS-Di-based co-dominant genotyping system and construction of a linkage map

**Fig. 3:**
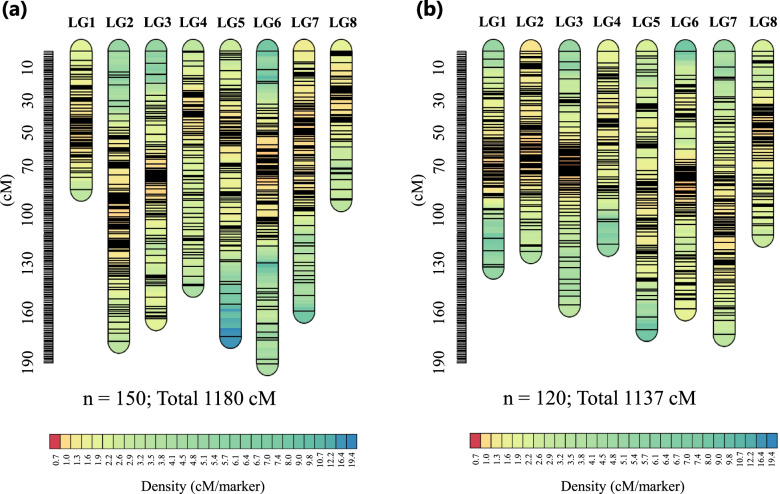
Genetic linkage maps of Crosses A and B_1. **a** Cross A; **b** Cross B_1. Black lines indicate marker positions and colours along each linkage group indicate marker density, with extremely dense regions appearing as black due to tightly clustered markers. LG, linkage group

To construct a genetic linkage map, we performed GRAS-Di analysis with 150 F_2_ progenies of Cross A (2019) and 120 F_2_ progenies of Cross B_1. Ordinary GRAS-Di genotyping software provided dominant markers and a small number of co-dominant markers in our data. Thus, we compared three genotyping systems: (1) GRAS-Di mapping–based genotyping with Buckwheat Genome DataBase (BGDB) [28] as a reference, (2) GRAS-Di mapping–based genotyping with ‘Dasha’ [29] as a reference, and (3) GRAS-Di co-dominant genotyping (GRAS-Di-CDG) in Cross A (Table 1). Because the total number of usable markers and average marker distance of the GRAS-Di-CDG system were better than those of both mapping-based genotyping systems, we used the GRAS-Di-CDG system to construct linkage maps in Crosses A and B_1. The number of linkage groups (LGs) matched the chromosome number of buckwheat in both crosses (Fig. 3). For Cross A, GRAS-Di-CDG estimated marker genotypes in 896 and 889 loci using a mixture of gamma or normal distributions, respectively; among them, 725 loci were redundant. For Cross B_1, GRAS-Di-CDG estimated marker genotypes in 1249 and 1062 loci, respectively, and 870 loci were redundant. The average accuracy of co-dominant genotyping based on a gamma distribution was 95.6% in Cross A (95 loci) and 93.8% in Cross B_1 (89 loci). The average accuracy based on a normal distribution was 67.7% in Cross A (192 loci) and 77.3% in Cross B_1 (112 loci). Because the accuracy was higher when a mixture of gamma distributions was used, we prioritised these estimates when we define the genotypes for markers redundant in estimation by gamma or normal distributions. To 84 (Cross A) and 44 (Cross B_1) co-dominant markers obtained by the GRAS-Di default, the GRAS-Di-CDG added 733 (Cross A) and 805 (Cross B_1) markers. We bridged each LG between Crosses A and B_1 on the basis of the markers with identical primer sequence (Table S1). There were 118 such markers, and no marker was mapped on any different LG (Fig. S3).

**Table 1:** Comparison of the GRAS-Di analysis with mapping-based genotyping systems and a GRAS-Di-CDG system

System	Mapping_BGDB	Mapping_Dasha	GRAS-Di-CDG
*Cross A (n = 150)*			
Mapping reference	BGDB_20contigs.fa	Dasha_20contigs.fa	–
Markers (dominant + co-dominant)	5551 (1^a^ + 5555)	5165 (1^a^ + 5164)	829 (1^a^ + 828)
*Filtering step with Rqtl*			
Removed duplicated markers	2994	2749	701
Removed distorted markers	888	857	678
Removed markers within 1 kb of each other	607	632	–
*Linkage map construction in Antmap*			
Removed markers > 20 cM from adjacent marker	532	567	666
Number of linkage groups (usable markers)	8 (529)	8 (558)	8 (666)
Map length (cM)	2051.8	2104.2	1179.8
Average marker distance (cM)	3.88	3.77	1.77

^a^Flower morphological marker (Pin or long-homostyle). This morphological marker was mapped as “Flower_type” in Fig. S3 and listed in Table S4

### QTL analysis for maturity time and flowering time

**Fig. 4:**
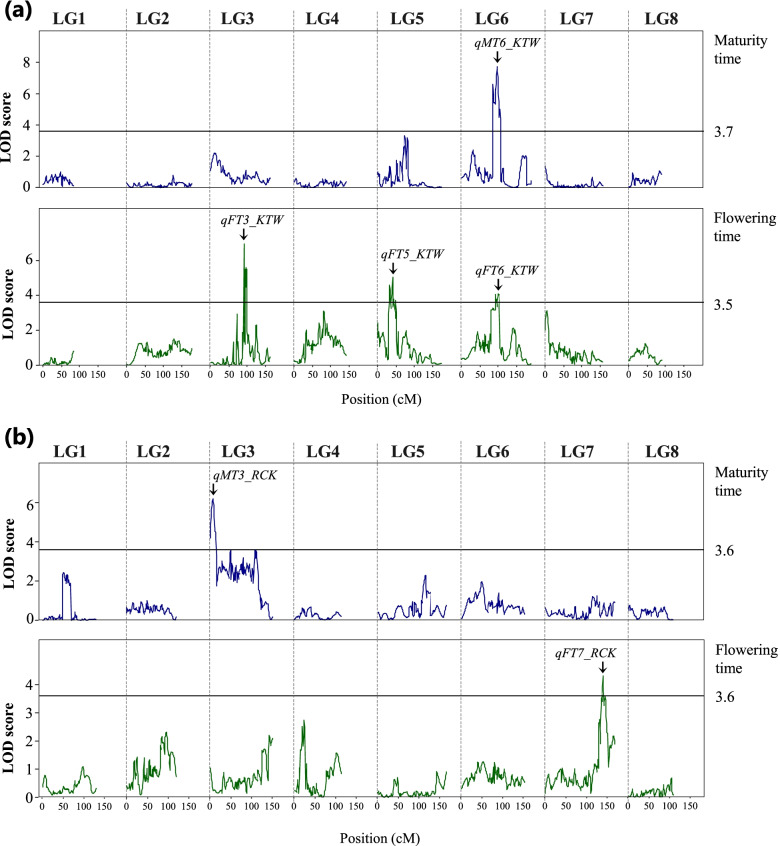
QTL plots in Crosses A and B_1. The LOD scores for maturity time and flowering time in **a** Cross A and **b** Cross B_1. The threshold of each QTL is indicated by a horizontal black line

Identified QTLs are shown in Table 2 and Fig. 4. In Cross A, one major QTL for maturity time (*qMT6_KTW*) was detected in LG6 and explained 21.0% of phenotypic variation. The KTW allele at *qMT6_KTW* conferred early maturity and may be partially dominant. Three QTLs for flowering time were detected in LG3 (*qFT3_KTW*), LG5 (*qFT5_KTW*), and LG6 (*qFT6_KTW*). The most effective of them, *qFT3_KTW*, explained 15.4% of phenotypic variation. In Cross B_1, one major QTL for maturity time was detected in LG3 (*qMT3_RCK*) and explained 20.5% of phenotypic variation. The RCK allele at *qMT3_RCK* conferred late maturity and may be dominant or partially dominant. One QTL for flowering time was detected in LG7 (*qFT7_RCK*) and explained 13.6% of phenotypic variation. These results indicate that maturity time and flowering time are regulated by different genetic mechanisms.

**Table 2:** Summary of detected QTLs

Populations	Trait	QTLs	LG	Closest marker (position, cM)	Peak position (cM)	LOD	Additive effect^a^	Dominant effect	R2 (%) ^b^
Cross A	Maturity time	*qMT6_KTW*	6	AMP0019203 – AMP0011313	96.6	7.74	−7.19	−3.28	21.0
				(93.2)–(96.6)					
	Flowering time	*qFT3_KTW*	3	Toyo0004322 – AMP0024401	91.7	6.96	−6.52	−0.03	15.4
				(91.3)–(91.7)					
		*qFT5_KTW*	5	AMP0000566 – AMP0023981	40.8	5.03	0.59	−0.5	11.1
				(39.8)–(40.8)					
		*qFT6_KTW*	6	AMP0011313 – AMP0026722	100.6	4.09	−0.48	−0.49	9.3
				(96.6)–(101.3)					
Cross B_1	Maturity time	*qMT3_RCK*	3	AMP0019836 – AMP0023439	6.1	6.20	4.67	5.76	20.5
				(6.1)–(11.2)					
	Flowering time	*qFT7_RCK*	7	AMP0005537 – AMP0029656	143.0	4.31	0.63	−0.89	13.6
				(140.9)–(143.0)					

^**a**^The values indicate that the effect is contributed by the alleles from KTW in Cross A and RCK in Cross B_1

^b^Percentage of total variation in marker association for each trait across the population explained by the QTL

### Association analysis of QTLs for maturity time

We first determined the QTL-linked scaffolds by local BLAST and developed sequence-tagged-site (STS) markers for *qMT6_KTW* and *qMT3_RCK* (Tables S2 and S3). In Cross A, plants homozygous for the KTW allele of *qMT6_KTW*_STS matured on average 7.4 days (2019) and 9.0 days (2020) earlier than those homozygous for the KSC7 allele (Table 3). In Cross B, plants homozygous for the RCK allele of *qMT3_RCK*_STS matured on average 6.7 days (2018; Cross B_2), 6.8 days (2019; Cross B_1), and 2.9 days (2020; Cross B_3) later than those homozygous for the KSC7 allele. Average maturity time values of heterozygous plants were almost the same as those of RCK-homozygous plants, suggesting that the RCK allele at *qMT3_RCK* behaved as dominant or partially dominant. In all crosses except Cross B_3, differences in average maturity time between plants homozygous for the marker genotypes were statistically significant (Table 3).

**Table 3:** Association between segregation of genotypes at DNA markers of QTLs and maturity time

Population (Year)	Marker name	Number of plants	Number of plants of each genotype ^*a*^			Maturity time (days after sowing) Mean ± standard deviation			*P* value ^*b*^		
			AA	AB	BB	AA	AB	BB	AA–AB	AB–BB	AA–BB
Cross A (2019)	*qMT6_KTW_ STS*	131	44	52	35	79.5 ± 6.5	82.2 ± 8.9	86.9 ± 11.4	0.30951	0.0437*	0.00102**
Cross A (2020)	*qMT6_KTW_ STS*	178	36	87	55	71.0 ± 5.1	78.2 ± 10.5	80.0 ± 10.1	0.00047***	0.5179	0.00005***
Cross B_1 (2019)	*qMT3_RCK_ STS1*	89	28	43	18	98.0 ± 8.3	99.4 ± 7.1	91.2 ± 9.4	0.75597	0.0013**	0.0159*
Cross B_2 (2018)	*qMT3_RCK_ STS1*	137	49	53	35	113.2 ± 11.0	111.4 ± 11.8	106.5 ± 10.1	0.70079	0.1065	0.0196*
Cross B_3 (2020)	*qMT3_RCK_ STS2*	150	36	75	39	94.5 ± 9.5	93.5 ± 9.6	91.6 ± 10.5	0.86219	0.5981	0.4074

^*a*^A, alleles from KTW or RCK; B, alleles from KSC7

^*b*^Analysed by Tukey–Kramer test

*P < 0.05, **P < 0.01, ***P < 0.001

## Discussion

### Detection of novel QTLs for maturity time

Understanding ecotype differentiation is essential for adaptive expansion and maximizing yield. Determination of buckwheat ecotype is based on the adaptability of the cultivar to the environment in each area, but the genetic analysis of ecotypes has focused only on photoperiod-dependent flowering time [17, 18]. In this study, we focused on maturity time as a trait that defines the ecotype. There was no strong correlation between flowering time and maturity time in our field experiments, and maturity time appeared to be more suitable to explain ecotype differentiation (Fig. 1 and Fig. S2). Thus, the differentiation between the summer and autumn ecotypes may be mainly defined by the ability to set seed and by maturity time, whereas flowering time is less relevant. To determine the genetic region that regulates maturity time, we developed GRAS-Di-CDG and used it to construct highly reliable linkage maps (Table 1 and Fig. 3). Using these maps, we detected one major QTL for maturity time from Cross A (*qMT6_KTW*) and one from Cross B_1 (*qMT3_RCK*) (Table 2 and Fig. 4).

The KTW allele at *qMT6_KTW* conferred early maturity (Tables 2 and 3). KTW was bred from ‘Botan-soba’, which is a cultivar in Hokkaido, the northern island of Japan [30]. Since only summer cultivation is possible in Hokkaido, summer ecotype traits—early seed set and maturity under LD conditions—are essential. The summer ecotype is considered to have differentiated from the autumn ecotype to adapt to northern areas [12, 31, 32]. Thus, *qMT6_KTW* might have contributed to adaptive expansion of ‘Botan-soba’ and its ancestor to northern areas. On the other hand, the RCK allele at *qMT3_RCK* conferred late maturity. RCK was bred from gamma ray–irradiated ‘Botan-soba’ populations at Ibaraki Prefecture (middle latitude of Japan) [33]. RCK has been selected for autumn cultivation during breeding, so it may have retained the late maturity allele. The summer ecotype has low yield under SD conditions, such as low-latitude areas or autumn cultivation; thus, *qMT3_RCK* may contribute to the fine-tuning of the growth period to increase yield under autumn cultivation.

Ecotype breeding has been conducted only by phenotypic selection, but it could be accelerated by genotype-based selection in allogamous common buckwheat. Our detected QTLs and the STS markers could accelerate ecotype breeding and expand buckwheat cultivation areas and seasons. In addition, the late-maturity QTL *qMT3_RCK* might be useful for fine-tuning the growth period to increase yield in low-latitude areas or autumn cultivation.

### Different genetic mechanisms regulate photoperiod-dependent flowering time and maturity time

We found no strong correlation between flowering time and maturity time in our field experiments (Fig. 1 and Fig. S2). Hara and Ohsawa [32] investigated photoperiodic sensitivity in two buckwheat cultivars, the autumn ecotype ‘Miyazaki-zairai’ and the summer ecotype ‘Botan-soba’, under photoperiods ranging from 12.0 to 15.5 h. Under the SD condition (12.0 h), flowering time of ‘Botan-soba’ and ‘Miyazaki-zairai’ were almost the same, whereas flowering time of ‘Miyazaki-zairai’ was significantly delayed than ‘Botan-soba’ under the LD conditions (14.5, 15.0, and 15.5 hr). However, ‘Miyazaki-zairai’ showed large genetic diversity (e.g., the range of flowering time was 26 to 90 days under 15.0 h photoperiod). These results indicate that differentiation between summer and autumn ecotypes can be evaluated from photoperiod-dependent flowering time, but for some individual plants flowering time does not reflect the ecotype, depending on their genotypes or cultivation conditions. The latter possibility is supported by the study by Michiyama and Hayashi [15], who reported that the most notable difference between ecotypes under summer conditions was in post-flowering development. Cultivars of the autumn ecotype continued to develop stems, leaves, and flower clusters without seed set, resulting in a significant delay in maturity time in comparison with the summer ecotype. These reports and our field experiments suggest that ecotype differentiation sometimes cannot be evaluated only from flowering time, and the ability to set seed and maturity time are more suitable to explain ecotype differentiation. This hypothesis suggests that different genetic mechanisms control flowering time, seed set, and maturity time. Indeed, we detected QTLs for flowering time and maturity time in different genetic loci except for *qFT6_KTW*, which may be the same QTL as *qMT6_KTW* (Table 2 and Fig. 4). Hara et al. [18] detected five QTLs for flowering time under SD conditions and six under LD conditions. We used markers around those QTLs [18] in Cross A and were able to map seven of them (Table S4). Of these, Fest_L0013_2 was mapped at 50.6 cM on LG3, 41 cM away from *qFT3_KTW* (91.7 cM). In Hara et al. [18], Fest_L0013_2 was located near *qFT12hL × E_1*, the most effective QTL for flowering time under SD conditions. The other six markers were not mapped close to any of the other QTLs in our study. These results suggest that different genetic mechanisms regulate maturity and flowering time in buckwheat. Because flowering time and maturity time are thought to be synchronized in many crops, adaptability has been evaluated on the basis of genetic mechanisms related to flowering time [34, 35]. However, our study demonstrates that flowering and maturity times are not synchronized in buckwheat, at least under natural LD conditions, and the genetic mechanism of maturation is important for adaptability. Guan and Adachi [21] reported that summer cultivation increase the proportion of abnormal embryo sacs after pollination and decrease the ability to set seed in the autumn ecotype. Nakamura and Nakayama [36] reported that sterility of the autumn ecotype under summer cultivation might be caused by incomplete development of the pistil. From these reports, it can be inferred that the ecotype of buckwheat is regulated by the normal development of floral organs and seed-set ability in a particular environment. If seed set is inadequate, flowering and vegetative growth will continue, and maturity time will be delayed, so the ability to set seed affects the timing of maturation. Thus, the post-flowering transition toward maturity may require a seed-set-enabling genetic mechanism, which likely differs from those required for flowering. To the best of our knowledge, there is no identified genes that regulate flowering, seed-set and maturity time in buckwheat. In this study, we detected QTLs for maturity time with GRAS-Di-markers. The reference genomes we used are divided into many scaffolds, and there is a possibility that GRAS-Di-markers do not cover all scaffolds around the QTLs. To predict the candidate genes, we searched homologous regions of fragments of *qMT6_KTW_STS* and *qMT3_RCK_STS1* with the Tartary buckwheat (*F. tataricum*) genome, which was developed to a pseudomolecule-level [37] (Table S5). The fragment of *qMT6_KTW_STS* could not determine homologous regions because the hit length and E value were almost the same among the top three hits. The homologous region of the fragment of *qMT3_RCK_ STS1* was Ft3:38,046,779-38,047,144. We searched predicted genes and their annotations within 200 kb up and down from the homologous region of *qMT3_RCK_ STS1* (Table S6). Thirty-six genes were identified in the vicinity of the homologous region of *qMT3_RCK_ STS1*, but there was no gene related to flowering or maturing response to photoperiod. So, further study will be needed to identify genes controlling each QTL and their function for seed-set and maturity time in common buckwheat.

Future re-domestication of orphan crops and neo-domestication of underutilized plants will require the elucidation of genetic mechanisms underlying not only flowering time but also maturity time for adaptability expansion.

### GRAS-Di-CDG system for construction of a high-density genetic map without a high-quality reference genome

We previously performed next-generation sequencing (NGS)-based bulked segregant analysis (NGS-BSA) with the Ion AmpliSeq targeted sequencing technology (Thermo Fisher Scientific, Waltham, MA, USA) to rapidly construct a genetic map and conduct QTL analysis [24]. In that study, we used two types of custom AmpliSeq marker sets: a target trait–linked marker set developed from NGS-BSA data and a genome-wide marker set. Although this system is efficient for constructing a linkage map, rapidly narrows down the QTL region, and detects many SNPs in this region, the usability of the AmpliSeq genome-wide marker set depends on the genetic structure of the parents.

The GRAS-Di analysis is more flexible in terms of the genetic structure of the parents due to its large number of markers amplified by random primers, but it provides mainly dominant markers [25]. To obtain co-dominant markers from GRAS-Di data, we developed a GRAD-Di-CDG system that re-estimates these markers from read-depth count distribution. Our GRAS-Di-CDG system provided genome-wide high-density co-dominant markers, and the total number of usable markers and the average marker distance were better than those obtained with a mapping-based genotyping system with BGDB or ‘Dasha’ (Table 1). Using markers provided by GRAS-Di-CDG, we constructed the genetic maps whose total length was 1180 cM in Cross A and 1137 cM in Cross B (Fig. 3). Both our linkage map included a larger number of usable markers (or loci) and had a smaller average marker distance between loci than previous maps, such as those constructed with 269 cleaved-amplified polymorphic sequences and insertion/deletion (indel) markers (752.5 cM) and an F_2_ population [18], AmpliSeq markers (550.1 cM) and an F_2_ population [24], and 346 loci (773.8 cM) or 410 loci (800.4 cM) provided by microarray analysis of an F_1_ pseudo-test cross [38]. GRAS-Di-CDG performed an additional estimation step for the co-dominant marker genotype based on a mixture of distributions of amplicon read counts. Because this additional estimation would increase error, we removed markers with low estimation accuracy on the basis of GRAS-Di default output data. These marker genotype data yielded many usable markers and highly reliable linkage maps (Table 1, Fig. 3 and Fig. S3). Thus, GRAS-Di-CDG provides an efficient way to obtain genome-wide co-dominant markers and rapidly construct a high-density linkage map without a high-quality reference genome. The use of these two systems for different purposes (e.g., NGS-BSA with targeted amplicon sequencing for qualitative traits or to obtain information on many SNPs around the QTL of the target trait, and GRAS-Di-CDG for quantitative or multiple traits) can efficiently promote the genetic analysis of buckwheat.

## Conclusions

In this study, we focused on maturity time as a trait that defines the ecotype. We developed GRAS-Di-CDG, used it to construct a high-density linkage map and detected major QTLs for maturity time. The allele of one QTL (*qMT6_KTW*) from KTW conferred early maturity, and the allele of another QTL (*qMT3_RCK*) from RCK conferred late maturity. To the best of our knowledge, this is the first report of QTLs for maturity time in buckwheat. These QTLs and STS markers may be useful to extend the cultivation area and to fine-tune the growth period of buckwheat to maximize yield.

## Methods

### Plant materials

To measure the variation in photoperiod response, 15 germplasms from Russia, Canada, China, Japan, France, Pakistan, Myanmar, and Brazil were used. The stock name and accession number of each germplasm are listed in Table S7. All plant materials are available in the NARO Genebank (https://www.gene.affrc.go.jp/about_en.php).

The SI system of buckwheat is controlled by a single genetic locus, *S*; thrum is heterozygous (*Ss*) and pin is homozygous recessive (*ss*). The SC line has a long homostyle (LH) controlled by a single allele, *S*^*h*^, in the dominance relationship *S* > *S*^*h*^ > *s* [39]. To develop F_2_ segregating populations, we used pin plants (*ss*) of KTW (early-maturing) and RCK (intermediate-maturing) as seed parents and the SC line KSC7 (*S*^*h*^*S*^*h*^) (late-maturing) as a pollen donor. KSC7 was developed from ‘Norin-PL1’, which was derived from an interspecific cross between common buckwheat and its SC wild relative, *F. homotropicum* [39, 40] (Fig. S4). Four segregating populations from independent crosses were developed: Cross A (KTW/KSC7), Cross B_1 (RCK_1/KSC7), Cross B_2 (RCK_2/KSC7), and Cross B_3 (RCK_3/KSC7). The F_2_ seeds of Cross A were divided into two and sowed in two seasons. The other F_2_ populations were sowed for one season (See *Field experiments* for detail).

The F_2_ progenies we used for the map construction and QTL analyses segregated the flower morphology as LH (*sS*^*h*^ or *S*^*h*^*S*^*h*^) and pin (*ss*) fitting to the expected ratio of 3:1 for a single dominant gene (Table S8).

### Field experiments

All 15 germplasms, each parental line, and F_2_ populations were grown in a field of the Institute of Crop Science, NARO, Tsukuba, Japan (latitude 36.027186, longitude 140.103022). The sowing dates were April 14, 2019 (germplasms), April 16, 2018 (Cross B_2), April 14, 2019 (Crosses A and B_1), and April 16, 2020 (Crosses A and B_3). Seeds were sown with a row length of 2 m, row space of 70 cm, and distance between plants of about 13 cm. Flowering time was recorded as DAS to first flowering. Maturity time was recorded as DAS to maturity of 80% of the seeds, as judged from their colour. The experiment ended at 122 DAS in 2018 (Cross B_2), and 112 DAS (germplasm) or 105 DAS (Crosses A and B_1) in 2019, and 105 DAS (Cross A) or 112 DAS (Cross B_3) 2020.

### GRAS-Di analysis and genotyping with markers around flowering time QTLs

The GRAS-Di technology was developed by Toyota Motor Corporation (Aichi, Japan) [25] and its Patent ID is P2018-42548A. This technology consisted of sample preparation using high concentration random primer, NGS and data analysis. The library of GRAS-Di is constructed by two sequential PCR steps; the first PCR primers included Illumina Nextera adaptor sequences plus 3-base random oligomers and the second included Illumina multiplexing 8-base dual index and P7/P5 adapter sequence. In this study, the F_2_ progenies of Cross A (*n* = 150) and Cross B_1 (*n* = 120), and their parents were used for GRAS-Di analysis. Genomic DNA was isolated from young leaves of each plant with a DNeasy Plant Mini Kit (Qiagen, Hilden, Germany). GRAS-Di analysis was performed under contract at Gene Bay, Inc. (Kanagawa, Japan). Each DNA sample was amplified with 12 random primers. Libraries were prepared as described in Ito et al. [41] and sequenced using the Illumina HiSeq 4000 platform. Markers were identified with GRAS-Di software v 1.0.4 (Toyota, Aichi, Japan). All usable marker sequences are listed in Table S4.

Using markers around QTLs for flowering time under SD and LD conditions [18], we performed genotyping in Cross A. Amplification with genomic DNA as a template was performed with the designed specific primers and ExTaq (TaKaRa, Shiga, Japan) as follows: 32 cycles at 94 °C for 30 s, 58 °C for 30 s, and 72 °C for 30 s. Amplification was confirmed by agarose gel electrophoresis. Primer sequences and restriction enzymes are listed in Table S2.

### Mapping-based genotyping

Both available reference sequences—the one in the BGDB (http://buckwheat.kazusa.or.jp/) [28] and ‘Dasha’ reference genome [29]—are divided into large numbers of scaffolds and require considerable computer memory and runtime for joint genotyping. To reduce the computational load, we combined these scaffolds into 20 segments as follows: (1) two custom reference sequences that contained only ≥1 kb scaffolds were developed as Out_1Kb_BGDB.fa and Out_1Kb_Dasha.fa with SeqKit v0.13.2 [42]; (2) these custom references were combined into 20 segments with split -p 20 in SeqKit v0.13.2, and BGDB_20contigs.fa and Dasha_20contigs.fa reference genomes were developed (Table S9).

For mapping, low-quality reads and adaptors were removed from GRAS-Di raw reads with fastp v.0.20.1 [43] using parameters -q 25, −n 5, −f 3, −F 3, and -l 30. The filtered reads were mapped with BWA-MEM2 v.2.2.1 [44] on BGDB_20contigs.fa and Dasha_20contigs.fa. Each BAM file was sorted and indexed using SamTools v.1.12 [45]. HaplotypeCaller in GATK v4.1.9.0 [46] was used with the --emit-ref-confidence and GVCF options to call variants in each individual. All gvcf files were merged using GATK GenomicsDBImport with the –intervals option (split 20 segments). GATK GenotypeGVCFs with the -all-sites option were used for joint genotyping. All VCF files that contained indels, low-quality SNPs, SNPs with more than two alleles, or more than 10 missing-count SNPs were excluded using Vcftools v 0.1.16 [47] with the following parameters: --remove-indels, −-min-meanDP 10, −-minQ 30, −-max-meanDP 50,000, −-min-alleles 2, −-max-alleles 2, and --max-missing-count 10.

### GRAS-Di-based co-dominant genotyping (GRAS-Di-CDG)

The default GRAS-Di dominant marker genotype was estimated according to the read depth data after adjustment with GRAS-Di software v 1.0.4. The read depth count in a locus with maternal-homozygous, heterozygous, and paternal-homozygous genotypes might show a trimodal distribution in a progeny population. To estimate the co-dominant marker genotype, we estimated a mixture of three gamma or normal distributions for read count data in each locus, using ‘gammamixEM’ and ‘normalmixEM’ functions in R-package “mixtools” [48]. The maximum number of iterations in EM algorithm was set to 5000, and markers that did not converge were removed from further analysis. The initial values of mixing proportions were set to 0.25, 0.50, and 0.25 to mimic the expected segregation ratio in an F_2_ population. When a mixture of normal distributions was assumed, the initial values of distribution means were set to 0, 50% quantile, and 75% quantile of the count data. When a mixture of gamma distributions was assumed, the pre-initial values of shape and scale parameters were set so that the distribution means were 0, 50% quantile, and 75% quantile of the count data, and the standard deviations were 1/3, 1/2, and 1/2 of the standard deviation of the count data. To obtain the initial values for EM algorithm calculations, 1.0 and 0.5 were added to the pre-initial shape and scale parameters, respectively. For other settings, the default parameters in each function were used. After estimation of the mixture of distributions, the marker genotype with the highest posterior probability was assigned to each observation.

In the default of GRAS-Di software genotyping system, co-dominant marker genotypes were determined for a small proportion of loci and were based on the paired dominant marker genotypes. Then, the accuracy of our co-dominant genotyping system was evaluated in two ways: (1) accuracy of co-dominant genotyping and (2) accuracy of dominant genotyping. Accuracy of co-dominant genotyping was calculated for markers with co-dominant marker genotypes estimated by the default system as the percentage of agreement with them. Accuracy of dominant genotyping was calculated for all markers as the percentage of agreement between the dominant genotypes estimated by the default system and those by our co-dominant genotyping system, in which estimated heterozygous genotypes were put into the correspond parental genotype.

Markers that showed greater than 95% consensus with the default dominant estimation were extracted. The markers that showed correct maternal and paternal genotypes in parents were used in the following analysis. We obtained three types of co-dominant marker genotype data (i.e., GRAS-Di default, gamma distribution estimation, and normal distribution estimation), which contained redundant information relative to each other. So, we extracted the marker genotypes according to the priority: (1) GRAS-Di default, (2) gamma distribution estimation, and (3) normal distribution estimation.

### Construction of a genetic linkage map and QTL analysis

Before linkage map construction, the data were preprocessed in R/qtl v 1.46–2 [49] as follows: (1) duplicate markers were identified that showed the same genotypes in all individuals except where values were missing, markers with the fewest missing values were selected, and duplicate markers were excluded; (2) markers with an abnormal genotype distribution (*P*-value < 0.001 in *X*^*2*^-test) were excluded. The genetic map was constructed in AntMap v 1.2 software with Kosambi Map function and 50 runs for locus ordering [50]. If the map distance between two adjacent SNPs was larger than 20 cM, the marker was excluded. The genetic linkage and densities was constructed with LinkageMapView v. 2.1.2 [51]. QTL analysis was performed in WinQTL Cartographer v. 2.5 software using the composite interval mapping model [52]. The significance thresholds of the log-likelihood (LOD) score were based on 1000 permutations (*P* = 0.05) and were as follows: Cross A, 3.7 for maturity time and 3.5 for flowering time; Cross B_1, 3.6 for both maturity time and flowering time.

### Development of sequence-tagged-site markers linked to QTLs

We converted QTLs around GRAS-Di markers to STS markers. We first performed local BLAST searches to find scaffolds matching these QTLs. A database for these searches that included the BGDB and ‘Dasha’ reference sequences was constructed from the Out_1Kb_BGDB.fa and Out_1Kb_Dasha.fa reference sequences (Table S9) using ncbi-blast-2.5.0+ [53]. Local BLAST was performed with SequenceServer v1.0.12 [54] (https://www.sequenceserver.com). We developed QTLs around STS markers on Fes_sc0001609.1 (for *qMT6_KTW*) and Fes_sc0023620.1 (for *qMT3_RCK*) (Table S3). Primer sequences and restriction enzymes are listed in Table S2.

## Supplementary Information

**Additional file 1: Table S1.** Bridge markers between Cross A and Cross B_1.

**Additional file 2: Table S2.** Primer information.

**Additional file 3: Table S3.** Results of local BLAST.

**Additional file 4: Table S4.** Marker information from GRAS-Di.

**Additional file 5 :Table S5.** Results of local BLAST with tartary buckwheat genome.

**Additional file 6: Table S6.** Gene list of tartary buckweat which located within 200 kb up and down from the homologous region of *qMT3_RCK_STS1*.

**Additional file 7: Table S7.** World buckwheat germplasms used in this study.

**Additional file 8: Table S8.** Segregation of flower morphology.

**Additional file 9: Table S9.** Summary of the reference databases.

**Additional file 10: Fig. S1.** Segregation of flowering time in F_2_ progenies of crosses between ‘Kitawase-soba’ (KTW) and ‘Kyukei SC 7’ (KSC7) (Cross A), and between ‘Ruchi-king’ (RCK) and KSC7 (Cross B). Arrows indicate mean values of flowering time in parents. DAS, days after sowing.

**Additional file 11: Fig. S2.** Scatter plot of flowering time and maturity time in F_2_ progenies. Black circles indicate plants that did not mature until the end of the cultivation period. *r*, Pearson correlation coefficients. DAS, days after sowing.

**Additional file 12: Fig. S3.** Bridging of the linkage maps of Crosses A and B_1. Marker sequences common between the two crosses are connected with dashed lines. LG, linkage group. GRAS-Di-CDG, the markers provided by the developed GRAS-Di-based co-dominant genotyping system. GRAS-Di-default, co-dominant markers provided by GRAS-Di analysis. Other, markers developed by Hara et al. [18]. Flowering Type, the dominant marker of floral morphology.

**Additional file 13: Fig. S4.** The schematic diagram of the breeding history of ‘Kyukei SC 7’ (KSC7) (a) and F_2_ populations (b). LH, long homostyle; SC, self-compatibility; SI, self-incompatibility; KTW, ‘Kitawase-soba’; RCK, ‘Ruchi-king’.

## Data Availability

All data generated or analysed during this study are included in the manuscript and its Additional files. The raw reads of GRAS-Di analysis of Cross A and Cross B_1 obtained in this study are available from the DDBJ/EMBL/NCBI under the accession number PRJDB13790 and PRJDB13783, respectively. The data used and/or analysed during the current study are available from the corresponding author on reasonable request.

## Abbreviations

BGDB: Buckwheat Genome DataBase
DAS: Days after sowing
GRAS-Di: Genotyping by random amplicon sequencing–direct
GRAS-Di-CDG: GRAS-Di co-dominant genotyping
KSC7: ‘Kyukei SC7’
KTW: ‘Kitawase-soba’
HTC: ‘Hitachi-akisoba’
Indel: Insertion/deletion
LD: Long-day
LG: Linkage group
LH: Long homostyle
LOD: Log-likelihood
NGS: Next-generation sequencing
QTL: Quantitative trait locus
RCK: ‘Ruchi-king’
SC: Self-compatibility
SD: Short-day
SI: Self-incompatibility
SNP: Single nucleotide polymorphism
STS: Sequence-tagged site
