# Supplementary material for: Genetic basis of maturity time is independent from that of flowering time and contributes to ecotype differentiation in common buckwheat (Fagopyrum esculentum Moench)
Source: BMC Plant Biol. 2022 Jul 21;22:353. doi: 10.1186/s12870-022-03722-6 (PMC9306078; doi:10.1186/s12870-022-03722-6)
Supplement: Supplementary file 10 — Additional file 10: Fig. S1. Segregation of flowering time in F2 progenies of crosses between ‘Kitawase-soba’ (KTW) and ‘Kyukei SC 7’ (KSC7) (Cross A), and between ‘Ruchi-king’ (RCK) and KSC7 (Cross B). Arrows indicate mean values of flowering time in parents. DAS, days after sowing. [file 12870_2022_3722_MOESM10_ESM.pptx]

## Slide 1
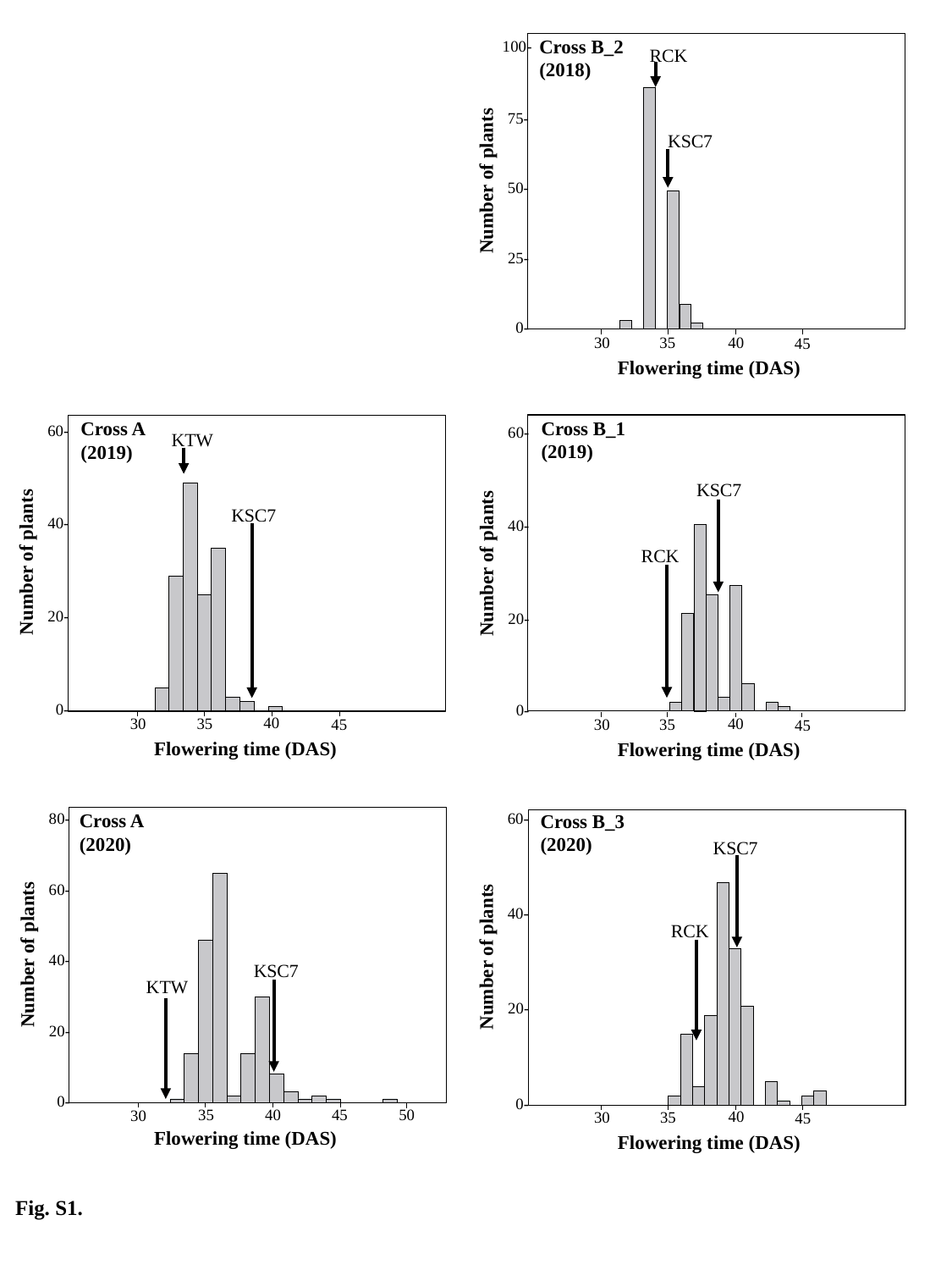

RCK
Cross B_2
(2018)
100-
75-
50-
25-
0-
Number of plants
KSC7
40
30
45
Flowering time (DAS)
35
Cross B_1
(2019)
Cross A
(2019)
KTW
60-
40-
20-
0-
Number of plants
60-
40-
20-
0-
Number of plants
KSC7
KSC7
RCK
40
30
45
Flowering time (DAS)
35
40
30
45
Flowering time (DAS)
35
80-
60-
40-
20-
0-
Number of plants
60-
40-
20-
0-
Number of plants
Cross A
(2020)
Cross B_3
(2020)
KSC7
RCK
KSC7
KTW
35
50
45
40
30
40
30
45
Flowering time (DAS)
35
Flowering time (DAS)
Fig. S1.
