# Supplementary material for: Genetic basis of maturity time is independent from that of flowering time and contributes to ecotype differentiation in common buckwheat (Fagopyrum esculentum Moench)
Source: BMC Plant Biol. 2022 Jul 21;22:353. doi: 10.1186/s12870-022-03722-6 (PMC9306078; doi:10.1186/s12870-022-03722-6)
Supplement: Supplementary file 11 — Additional file 11: Fig. S2. Scatter plot of flowering time and maturity time in F2 progenies. Black circles indicate plants that did not mature until the end of the cultivation period. r, Pearson correlation coefficients. DAS, days after sowing. [file 12870_2022_3722_MOESM11_ESM.docx]

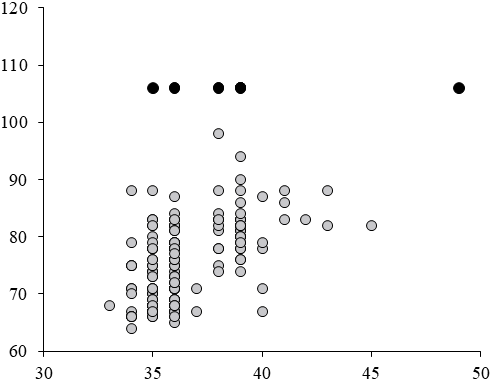


**Flowering time (DAS)**

**Maturity time (DAS)**

**Cross A**

**(2020)**


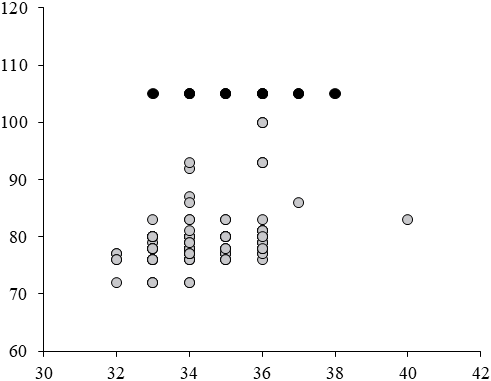


**Flowering time (DAS)**

**Maturity time (DAS)**

**Cross A**

**(2019)**


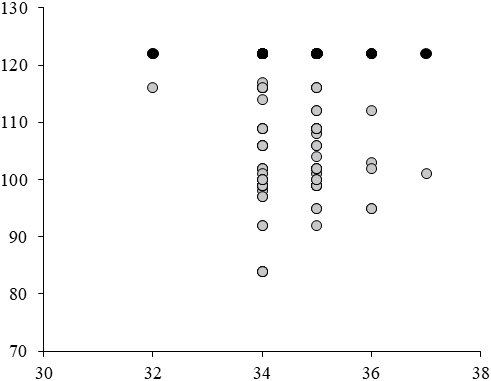


**Flowering time (DAS)**

**Maturity time (DAS)**

**Cross B_2**

**(2018)**


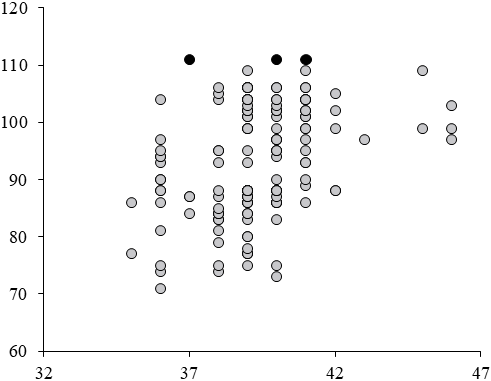


**Flowering time (DAS)**

**Maturity time (DAS)**

**Cross B_3**

**(2020)**


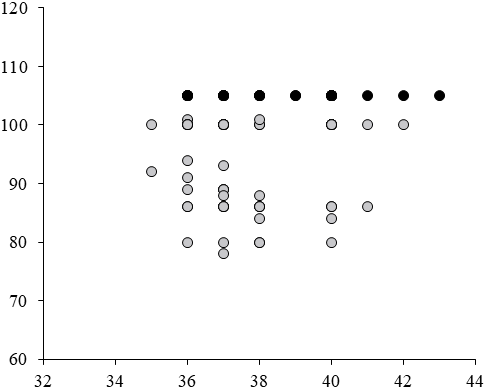


**Flowering time (DAS)**

**Maturity time (DAS)**

**Cross B_1**

**(2019)**

**Fig. S2.**

50

***r* = 0.392**

***r* = 0.506**

***r* = 0.165**

***r* = 0.365**

***r* = -0.08**
