# Supplementary material for: Genetic basis of maturity time is independent from that of flowering time and contributes to ecotype differentiation in common buckwheat (Fagopyrum esculentum Moench)
Source: BMC Plant Biol. 2022 Jul 21;22:353. doi: 10.1186/s12870-022-03722-6 (PMC9306078; doi:10.1186/s12870-022-03722-6)
Supplement: Supplementary file 12 — Additional file 12: Fig. S3. Bridging of the linkage maps of Crosses A and B_1. Marker sequences common between the two crosses are connected with dashed lines. LG, linkage group. GRAS-Di-CDG, the markers provided by the developed GRAS-Di-based co-dominant genotyping system. GRAS-Di-default, co-dominant markers provided by GRAS-Di analysis. Other, markers developed by Hara et al. [18]. Flowering Type, the dominant marker of floral morphology. [file 12870_2022_3722_MOESM12_ESM.docx]

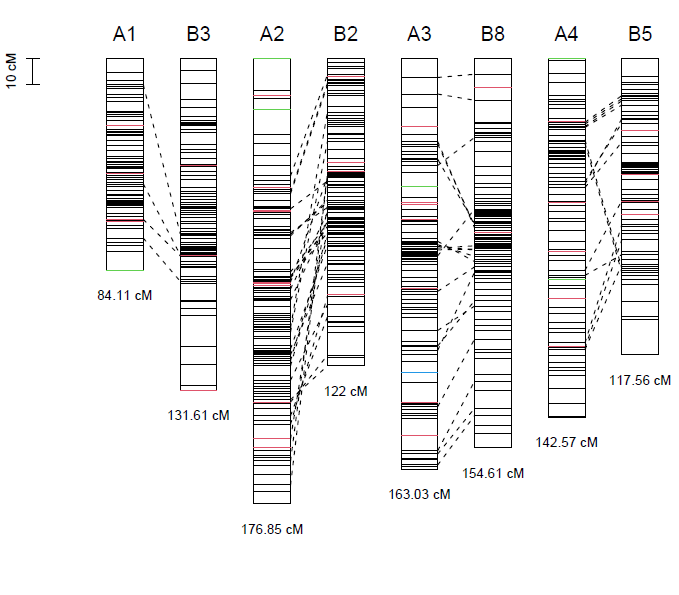


LG1

A

B

LG2

A

B

LG3

A

B

LG4

A

B


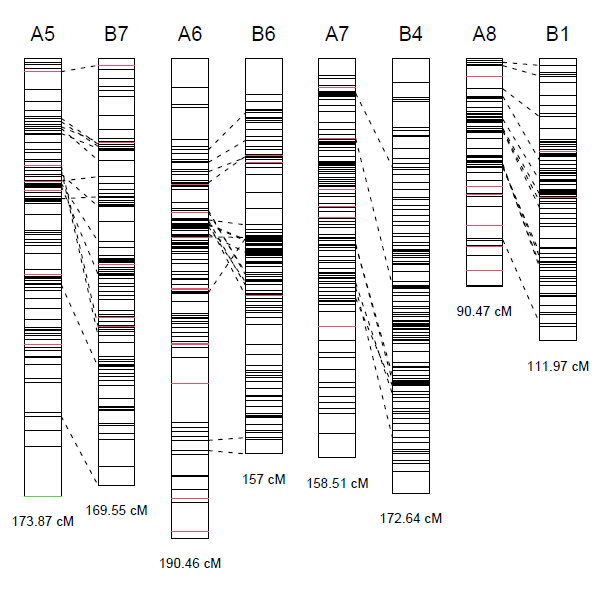


LG5

A

B

LG6

A

B

LG7

A

B

LG8

A

B

**Fig. S3.**

10 cM

84.1 cM

131.6 cM

176.9 cM

163.0 cM

154.6 cM

142.6 cM

117.6 cM

173.9 cM

169.5 cM

190.5 cM

172.6 cM

90.5 cM

112.0 cM

157.0 cM

158.5 cM

122.0 cM

◆ GRAS-Di-CDG

◆ GRAS-Di-default

◆ Other

◆ Flower_type
