# Supplementary material for: Genetic basis of maturity time is independent from that of flowering time and contributes to ecotype differentiation in common buckwheat (Fagopyrum esculentum Moench)
Source: BMC Plant Biol. 2022 Jul 21;22:353. doi: 10.1186/s12870-022-03722-6 (PMC9306078; doi:10.1186/s12870-022-03722-6)
Supplement: Supplementary file 13 — Additional file 13: Fig. S4. The schematic diagram of the breeding history of ‘Kyukei SC 7’ (KSC7) (a) and F2 populations (b). LH, long homostyle; SC, self-compatibility; SI, self-incompatibility; KTW, ‘Kitawase-soba’; RCK, ‘Ruchi-king’. [file 12870_2022_3722_MOESM13_ESM.docx]

**(a)**

**“Botansoba“**

**[Pin-SI (*ss*)]**

**×**

***F. homotropicum***

**[LH-SC (*S^h^S^h^*)]**

Embryo rescue

**F_1_ [LH-SC (*sS^h^*)]**

Select SC

**F_4_ [LH-SC (*S^h^S^h^*)]**

**“Botansoba“**

**[Pin-SI (*ss*)]**

**×**

Select SC and Non-shattering

**“Norin-PL1“ (B_1_F_11_)**

**[LH-SC (*S^h^S^h^*)]** (Matsui et al., 2008)

**×**

**“Hitachi-akisoba“**

**[Pin-SI (*ss*)]**

**F_1_ [LH-SC (*sS^h^*)]**

**×**

**“Asahimura-Zairai 3“**

**[Pin-SI (*ss*)]**

Select SC

**“KSC7” (F_13_)**

**[LH-SC (*S^h^S^h^*)]**

**(b)**

**“KTW” or “RCK“**

**[Pin-SI (*ss*)]**

**×**

**“KSC7”**

**[LH-SC (*S^h^S^h^*)]**

**F_1_ [LH-SC (*sS^h^*)]**

Selfing

**F_2_**

**[Pin-SI (*ss*), LH-SC (*sS^h^*), LH-SC (*S^h^S^h^*)]**

**Fig. S4.**
